# Mucin Production and Mucous Cell Metaplasia in Otitis Media

**DOI:** 10.1155/2012/745325

**Published:** 2012-05-22

**Authors:** Jizhen Lin, Per Caye-Thomasen, Tetsuya Tono, Quan-An Zhang, Yoshihisa Nakamura, Ling Feng, Jianmin Huang, Shengnan Ye, Xiaohua Hu, Joseph E. Kerschner

**Affiliations:** ^1^Department of Otolaryngology Head and Neck Surgery, University of Minnesota, 2001 6th Street SE, Minneapolis, MN 55455, USA; ^2^Department of Otolaryngology Head and Neck Surgery, University of Copenhagen, 2900 Copenhagen, Denmark; ^3^Department of Otolaryngology Head and Neck Surgery, University of Miyazaki, Miyazaki 889-1692, Japan; ^4^Department of Otolaryngology Head and Neck Surgery, Xi'An Jiaotong University, Xi'an 710004, Shaanxi, China; ^5^Department of Otolaryngology Head and Neck Surgery, Nagoya City University, Nagoya 467-8601, Japan; ^6^Department of Otolaryngology Head and Neck Surgery, Fujian Medical University, Fuzhou 350001, China; ^7^Department of Otolaryngology Head and Neck Surgery, Wisconsin Medical College, Milwaukee, WI 53201, USA

## Abstract

Otitis media (OM) with mucoid effusion, characterized by mucous cell metaplasia/hyperplasia in the middle ear cleft and thick fluid accumulation in the middle ear cavity, is a subtype of OM which frequently leads to chronic OM in young children. Multiple factors are involved in the developmental process of OM with mucoid effusion, especially disorders of mucin production resulting from middle ear bacterial infection and Eustachian tube dysfunction. In this review, we will focus on several aspects of this disorder by analyzing the cellular and molecular events such as mucin production and mucous cell differentiation in the middle ear mucosa with OM. In addition, infectious agents, mucin production triggers, and relevant signaling pathways will be discussed.

## 1. Introduction

Otitis media (OM) is characterized by the production of mucins in the middle ear mucosa. There are abundant mucous cells (goblet cells) in the inflamed middle ear mucosa whenever there is inflammation in the middle ear cavity [1–7]. In a typical case of purulent OM or acute bacterial middle ear infection, one thing an ENT clinical doctor would notice is mucus, together with pus, in the middle ear cavity and/or the external canal if the ear drum is perforated. In a typical case of chronic OM, more often than not, otolaryngologists would observe slimy substance or sticky mucus, like a rubble band, accumulated in the middle ear cavity. Similarly, one would see the same situation in the chronic mastoiditis. There is always mucus production and accumulation when there is an infection in the middle ear and mastoid mucosal system. This excessive mucous production interferes with mucosal function and plays a negative role in recovery of middle ear function and, therefore, contributes to the development of chronic OM. Many ENT doctors consider mucous cell metaplasia/hyperplasia chronic, irreversible, and intractable clinically.

**Figure 1:**
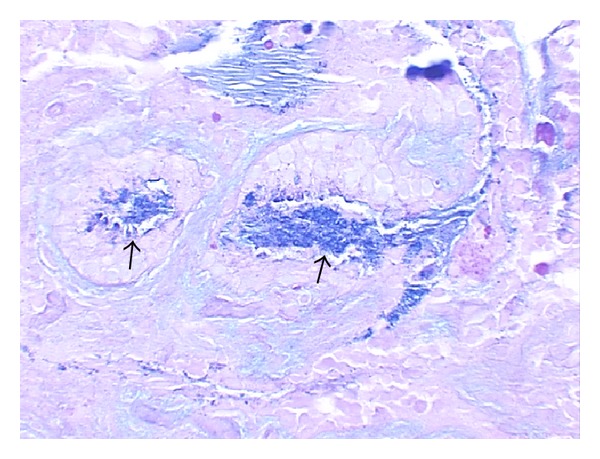
Mucous cell metaplasia/hyperplasia is identified with AB-PAS stain in a piece of middle ear mucosa (from a chronic OM patient) which contains abundant mucous cells (arrows, deep blue areas) and accumulated mucin glycoproteins in the central area of the gland-like structures. Amplification, ×20.

 Mucous cell metaplasia/hyperplasia is a pathological term that describes an increased mucous cell population or density in the upper respiratory mucosa upon infections and/or other stimuli. It is usually determined by histochemical examination with Alcian Blue-Periodic Acid Schiff (AB-PAS) stain which identifies large molecular weight glycoproteins (i.e., macroglycoconjugates or macroglycoproteins) in a cell. These macroglycoconjugates are heavily glycosylated proteins (mucins) which are a family of glycoproteins up to 20 members. A mucin glycoprotein typically consists of 10–15% protein back bones and up to 90% sugar side chains by mass [8, 9]. Mucins, together with mucin chaperones such as trefoil factors, are tightly packed into mucous granules that are positive for AB-PAS stain (Figure 1).

In the middle ear, mucous cell metaplasia/hyperplasia is a cardinal pathology in response to middle ear inflammatory and/or immune reactions [5]. Similar responses frequently occur in the airways in response to harmful insults [10]. In the lung, mucous cell metaplasia/hyperplasia can be life threatening due to its capability to block airway lumen by secreting a large amount of mucus [9, 11–15]. Particularly, this condition is referred to as chronic obstructive pulmonary diseases (COPDs). Cigarette smoking is the leading cause of COPD by stimulating mucus production of goblet cells.

 The reason for mucous cell metaplasia/hyperplasia is multifactorial [16]. External stimuli such as chronic microbiological infections, long-term exposure to other irritants such as cigarette smoking, air pollution, and certain gases such as SO_2_ can trigger mucous cell metaplasia/hyperplasia in the respiratory tract mucosa [17, 18]. Clinically, bacterial infection in the middle ear is the most common etiology. In children, the most common bacterium involved in mucous cell metaplasia/hyperplasia is *S. pneumococcus* and its metabolites. Indeed, OM with mucoid effusion is more frequently seen in child cases than adult ones [19]. Experimentally, pneumococcal infection in the middle ear of rats is prone to the development of mucous cell metaplasia/hyperplasia.

**Figure 2:**
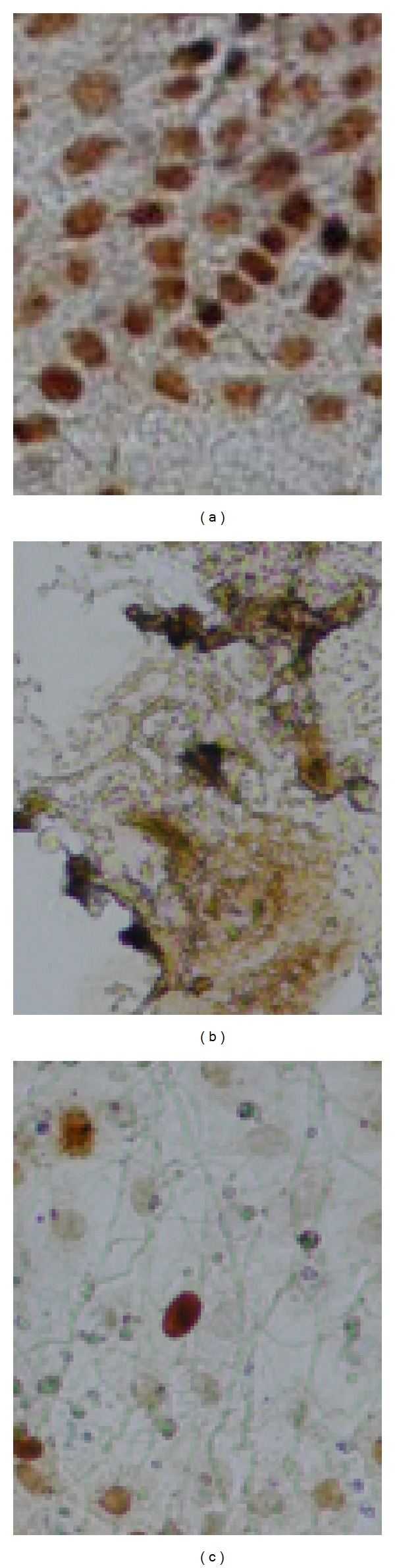
Pneumococcal cell wall component peptidoglycan-polysaccharides (PGPSs) stimulate the proliferation (a) of rat middle ear epithelial cells whereas endotoxin (LPS) causes the death of the cells (b) compared with carrier treated cells (c). Cells were incubated with PGPS at 20 ng/mL for 6 hours and stained with PCNA antibody. Brown in color indicates cell nuclei with positive PCNA antigen.

 In contrast, *H. influenza* tends to develop fibrotic disorders than mucous cell metaplasia/hyperplasia in animal experimental OM models [20, 21]. Biologically, peptidoglycan polysaccharides (PGPS, metabolites of *S. pneumonae*) stimulates epithelial proliferation whereas endotoxin (metabolites of *H. influenza*) promotes epithelial cell death in cultured middle ear epithelial cells, causing necrosis or apoptosis of the middle ear epithelial cells (Figure 2). This may explain why *S. pneumoniae* tends to cause mucous cell metaplasia/hyperplasia. However, both Gram-positive and -negative bacteria are potent inducers of mucous cell metaplasia/hyperplasia.Tos and Caye-Thomasenobserved an increased goblet cell density in acute OM, variable by causative pathogen [22].

 It is well recognized that mucous cell metaplasia/hyperplasia occurs in the acute event of middle ear infection but continues to deteriorate when there are recurrent or chronic OM. It is difficult to fully restore the mucous cell population or density back to the baseline level once mucous cell metaplasia/hyperplasia occurs in the middle ear mucosa. An episode of middle ear infection always adds some goblet cells to the mucosa even the infection even is long gone [22, 23].

Under normal conditions, few goblet cells exist in the middle ear cavity. These goblet cells are usually located in the orifice of the Eustachian tube, the partial promontory, and hypotympanic areas, so called “ciliated track.” Goblet cells increase dramatically following an acute middle ear infection or an acute onset of chronic OM. These proliferated goblet cells are all over the entire middle ear mucosa, not limited to the ciliated tract. Unlike ordinary epithelial cells, goblet cells are an integral part of the innate immune system. Therefore, maintenance of a stable goblet cell population in the upper respiratory tract including the middle ear epithelium is very important. It is observed that goblet cells are protected from apoptosis by a transcription factor called Id1 which keeps goblet cells alive on a long-term basis (approximately months). That is the reason why mucous cell metaplasia/hyperplasia remains in the diseased mucosa for a while (weeks to months after infections are dissolved).

 Clinically, the disease mechanism for mucous cell metaplasia/hyperplasia is not clear, but perceivably it involves persistent stimuli in the middle ear mucosa. Without repeated stimuli of bacteria or bacterial metabolites, mucous cell metaplasia/hyperplasia does not occur. Under normal conditions, transient stimuli do stimulate the differentiation of mucous cells in the middle ear mucosa, but the action does not last for too long. For example, injection of middle ear pathogens into the rat middle ear cavity induces transient mucous cell metaplasia/hyperplasia which lasts for weeks to months and then gradually subsides [24]. In other words, mucous cell metaplasia/hyperplasia occurs because there are chronic or recurrent stimuli persistently in the middle ear mucosa.

 However, the stimuli may be derived from chronic infections and sometime just from bacterial metabolites. One example is OM with mucoid effusion; frequently, there is no active infection in the middle ear cavity. It is well recognized that some dead bacterial cell wall fragments or metabolites are sufficient to keep the middle ear mucosa in a state of mucous cell metaplasia/hyperplasia.

## 2. Mucin Dysregulation in OM

 The first thing one would notice is that mucus is accumulated in the middle ear cavity when mucous cell metaplasia/hyperplasia occurs in the middle ear mucosa. It is frequently referred to as “glue ear" when the tympanic membrane is intact and mucins are accumulated in the middle ear cavity. In chronic OM or postsurgery patients, it is often referred to as “wet ear” in an open middle ear cavity because of continuous production of mucins in the diseased middle ear mucosa and persistent secretion into the surface of the middle ear mucosa. It has long been speculated that mucin upregulation is the first step for the epithelial cells to become mucous cells.

 Initially, those epithelial cells, rich in mucin production, remain nonmucous in terms of phenotypes, rather, mucin-producing cells. Mucin-producing cells are not necessarily mucous cells. In the upper respiratory tract, ciliated cells also produce limited amount of mucins, membrane-bound or secreted. Anyway, these mucin-producing cells by definition are not mucous cells, but they have the potential to become mucous or goblet cells if mucin production is further upregulated to a point at which cells produce only mucins and mucin chaperones but no others. If the origin of these cells is from ciliated cells, this is biologically called transdifferentiation of mucous cells.

 Under normal conditions, the origin of mucous cells is from epithelial stem cells above the basement membrane [25, 26]. Transdifferentiation occurs because there is an emergent need of mucous cells to secret mucins to discharge invading microorganisms. That is part of the innate immune response. In such a cellular crisis, there is no sufficient time for normal epithelial stem cells to respond and differentiate into mucous cells step by step. It has been noted that under normal conditions, the middle ear mucosa is not populated with mucous cells because of relatively clean and safe environments.

 It has been known that approximately 20 mucin genes have been identified, and 12 out of these genes have been shown to be expressed in the respiratory tract [9]. In terms of mucin(s) being upregulated, it is becoming clear that MUC5B is the predominant one. The recent work done by Preciado et al. has proved that MUC5B is abundant in the majority of chronic OM patients [5, 27, 28]. This is considered unusual because MUC5B is a major mucin expressed in submucosal glands (mucous cells) not in epithelial mucous cells in the entire upper respiratory tract. Occasionally, MUC5AC may be upregulated in OM [28].

**Figure 3:**
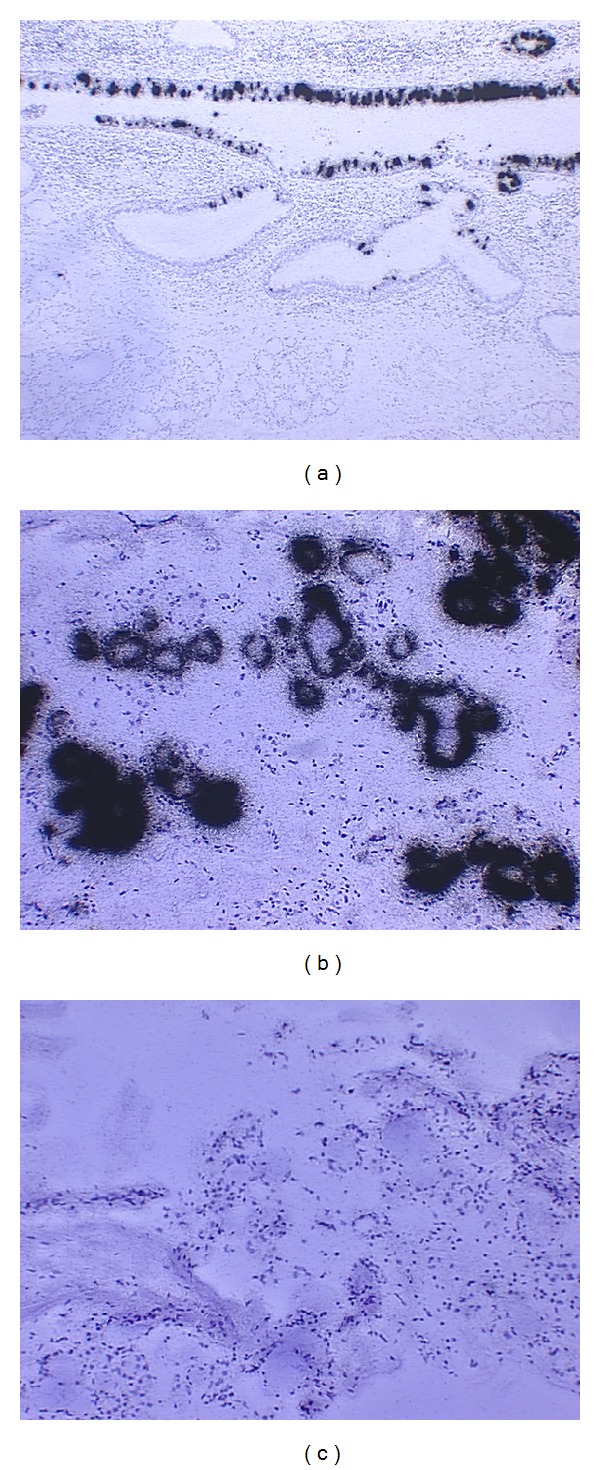
Mucins MUC5AC and MUC5B are two predominant mucins in the middle ear and Eustachian tube areas. (a) MUC5AC is typically expressed in the epithelial cells of the Eustachian tube. (b) MUC5B is predominantly expressed in the mucous cells of submucosal glands. (c) Control for in situ hybridization in (a) and (b). Note: Black dots indicate the mRNA transcripts for MUC5AC or MUC5B.

 From this viewpoint, one would argue that chronic OM triggers gland-like mucous cell differentiation in response to middle ear infections [29]. Indeed, in the surgical samples with chronic OM or mastoiditis, it has been observed that gland-like structures with abundant mucous cells are pathologically identified in the middle ear and mastoid area [30]. Gland-like structures are often observed in chronic OM patients (referred to as Figure 1). Consistent with this, in situ hybridization clearly demonstrated that MUC5AC-positive mucous cells are populated in the upper respiratory epithelium including the Eustachian tube whereas MUC5B-positive mucous cells are populated in the mucous glands of the upper respiratory epithelia (Figure 3).

 In contrast, middle ear epithelial cells are negative for the MUC5AC mRNA transcripts but spotty MUC5B mRNA transcripts are identified [4, 5]. This tells us that a transitional process of mucin members from the airway to the middle ear cavity occurs. This observation was confirmed recently by Preciado et al. that MUC5B mucin is predominant in chronic OM patients [27, 28]. However, other mucins such as MUC2 may be involved in middle ear mucus of animal models [31, 32], but their amount is limited or undetectable in humans if any [4, 5]. It has been noted that mucin quantification and comparison is notoriously difficult and at the best semiquantitative. This is because of the high level of glycosylation which represents a posttranslational modification.

 In a mucous cell, soluble mucins are secreted onto the cell surface under the direction of trefoil factors [33]. Soluble mucins expressed in mucous cells of the Eustachian tube epithelium include MUC5B and MUC5AC under normal conditions [4] whereas mucin expressed in mucous cells of the middle ear epithelium under chronic OM conditions is predominantly MUC5B in humans [4, 34] and Muc2 and Muc5ac in rodents [31, 35]. MUC4 and MUC1 membrane-bound mucins are involved in OM but not major ones. MUC5AC, MUC5B, MUC2, and MUC6 are known to be clustered at a locus on chromosome 11p15.5 in human [36], bearing similar properties and functions of soluble mucins. MUC5B in humans responds to chronic stimuli [28, 34], while Muc2 in rodents responds to acute stimuli such as inflammatory cytokine tumor necrosis factor alpha (TNF*α*) [37].

 As mentioned above, there are approximately 20 mucin genes currently being identified from the human body. They are either secretory mucins or membrane-bound mucins. The former is a class of mucins that are synthesized as monomers and then assembled into dimers or trimers in a head-to-head or a head-to-tail manner to form polymers. These mucins comprise mucous networks together with TFFs and spread around after secretion to the surface for protection of mucosal epithelial cells. On a smear, one can clearly observe mucous strings and witness their polymerization of soluble mucins.

**Figure 4:**
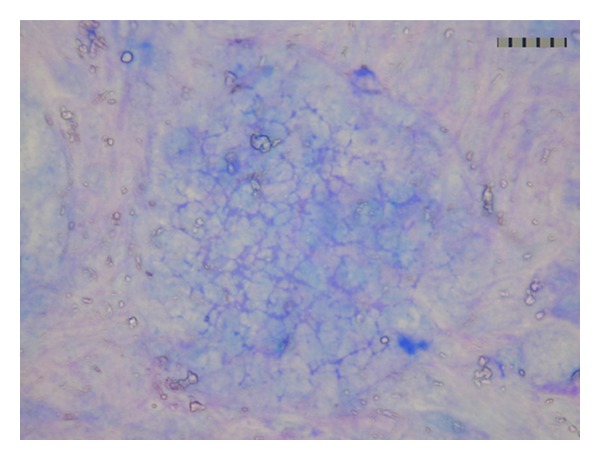
Mucous strings are networked substances under a microscope. Mucous granules are released from cultured mucous cell clone derived from HT-29, and mucous strings are stained blue on the cultured cell surface by AB-PAS. bar 50 *μ*m.

**Figure 5:**
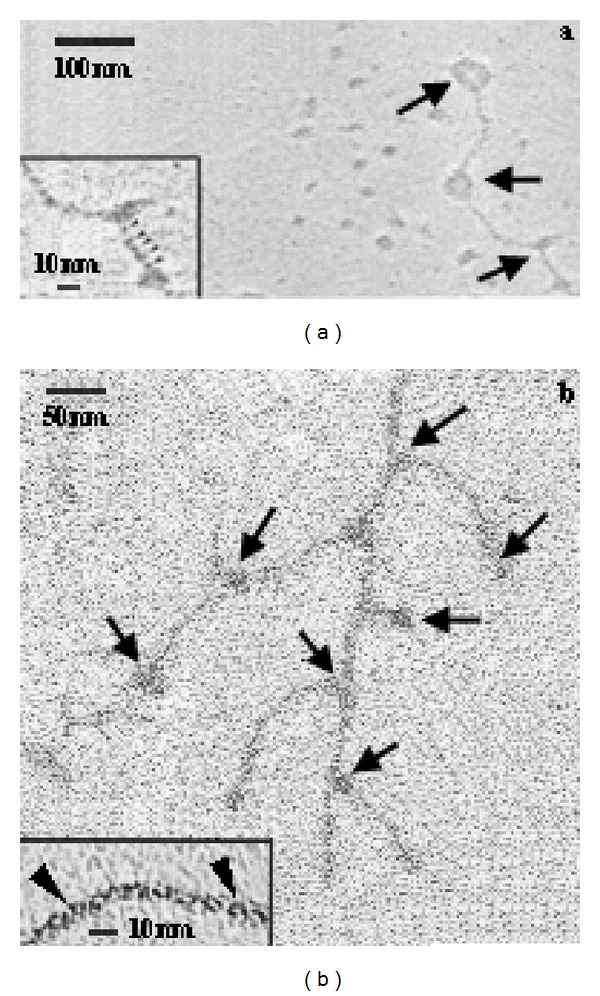
Mucin monomers are linked in a head-to-head manner in a mucous gel, which is a basis for forming a mucous gel. The inserts in (a) and (b) represent amplifications of a head-to-tail linkage (a node-like structure, (a)) or a mucin backbone (a twisted rope-like structure, (b)).

 Under a microscope, mucous strings are networked substances (Figure 4). Under an electromicroscope, mucin strings are linked head-to-head, forming polymers (Figure 5). Representative secretory mucins are MUC2, MUC5AC, MUC5B, MUC6, and so forth. The secretory mucin lacks a transmembrane domain and is, thus, doomed to be secreted. Membrane-bound mucins are a class of glycoproteins which are anchored directly to the bilayer of the cell membrane and prevent the epithelial cells directly from contacting with bacteria, viruses, or other particles. Representative membrane-bound mucins are MUC1, MUC3, MUC4, MUC7, and so forth.

## 3. Mucus Gel Composition in OM

 Mucus is mainly comprised of mucins [4, 5, 38–41] which impart viscous elastic and gel-forming properties to mucus [11–15]. The major mucins participate in the mucus gel and are secretory (soluble) mucins because of their water-soluble property. These soluble mucins are synthesized in mucous cells, stored in mucous granules, and then secreted onto the surface of epithelial cells by a regulated exocytosis which requires an external signal, a specific sorting signal on the vesicles, a clathrin coat, and an increase in intracellular calcium.

Occasionally, membrane-bound mucins such as MUC4 may participate in the mucus of middle ear effusion [5, 42] because these membrane-bound mucins are truncated by enzymes from bacteria and/or inflammatory cells and released into middle ear effusions. Membrane-bound mucins are biologically designed for cellular adhesion, pathogen binding/shielding, surface protection, and signal transduction [43, 44]. In addition, water, ions, lipids, and proteins such as mucin chaperones are integral parts of the mucus.

 In a mucin, sugar side chains (glycans, both *O*-glycans and *N*-glycans) can make up as high as 90 percent of the total protein by mass. *O*-glycosylation is a major part of mucin biosynthesis and requires an N-acetylgalactosaminyl peptidyltransferase. It occurs in the central region of a mucin molecule where there are numerous tandem repeats (typically doszens to several hundred). *N*-glycosylation typically occurs in the both end regions [9]. Recently, a newly characterized linkage of C-mannose to tryptophan (i.e., *C*-mannosylation) was identified in MUC5AC and MUC5B mucins synthesized *in vitro* [45]. Usually, mucin glycans contribute to the viscosity of mucins. The more sugar side chains a mucin contains, the more sticky or viscous a mucin is. Sugar side chains of a mucin contribute to the gel properties of mucus. Not only do mucins serve the function of protection, but they also act as lubricants and are integral structural components of the mucociliary transport system. Functionally, the sugar side chains act as receptors on cell surfaces that wrap invading microorganisms.

**Figure 6:**
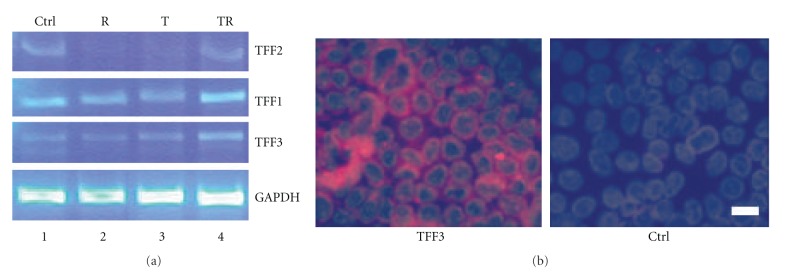
Mucin chaperones are inducible in cultured mouse middle ear epithelial cells. (a) TFF1, TFF2, and TFF3 are barely detected by reverse transcription polymer chain reaction (RT-PCR) in cultured mouse middle ear epithelial cells and are induced by TNF*α* and RA for two weeks (TR). (b) TFF3 is induced by TR treatment for two weeks as judged by immunohistochemistry. Ctrl, control; bar = 5 *μ*m.

 Main mucin chaperones are trefoil factors (TFFs) [33] which are represented by three members: TFF1(pS2), TFF2(hSP), and TFF3(ITF) [46]. Among them, TFF3 is expressed predominantly in the mucous cells of the small and large intestine. Under diseased conditions, all the TFF members can be expressed [47]. We have recently confirmed the expression of TFFs in mouse middle ear epithelial cells (Figure 6). In the human gut, TFFs and mucins are frequently coexpressed in mucous cells in a closely related manner. TFF1 with MUC5AC, TFF2 with MUC6, and TFF3 with MUC2 [48]. Other mucin chaperones include beta-defensins [49], secretory IgA [50], and lactoferrin [51], which also deserve attention.

 It is not clear whether the same relationship exists in the middle ear mucous cells. The TFF motif contains a hydrophobic binding pocket that could represent a binding site for sugar side chains of mucins, probably the oligomerisation domain (also called cysteine rich von Willebrand factor VWF/domain) of mucins, a common domain to all mucins, located in C- or N-terminal domains that permits disulfide bond to be formed between adjacent mucins. With addition of TFFs to mucins plus mucin itself multimerisation, the end result is the formation of a mucus gel with high viscosity [5, 52].

## 4. Mucoid Effusion in OM

 One of the manifestations of mucous cell metaplasia/hyperplasia is accumulation of mucoid effusion in the middle ear cavity [4, 29, 34, 53–57]. It is natural, on one hand, that mucus accumulated in the middle ear cavity if the Eustachian tube is not functional. On the other hand, excessive production of the mucus itself can cause the accumulation of mucus in the middle ear cavity even though the Eustachian tube is functional. The reason is that the viscosity of mucus secreted by the middle ear mucous cells, especially MUC5B mucin, is very high. MUC5B is one of the most viscous mucins because of its size and capability to form multiple molecules head-to-head as shown in previous reports [4, 5]. It is known that mucins are linked together by head-to-head or head-to-tail in a dimer or trimer manner. Other nonmucin molecules may form noncovalent interactions with mucins. The detailed linkage between individual mucins in the intestine for secreted mucins has been proposed as linear-polymer (N-terminal dimerization) or N-terminal-trimer netwok (N-terminal trimerization) models by McGuckin and coworkers [15]. While MUC5B mucins are chained together in a long string or network [4, 5, 15], this makes middle ear mucus very viscous and difficult to discharge.

 One may argue that the dysfunction of the Eustachian tube or the physical obstruction of the Eustachian tube (ETO) may also cause mucous cell metaplasia/hyperplasia. This issue has been studied recently in our laboratory and others. We have shown that ETO alone mainly induces accumulation of serous fluid in the middle ear cavity but rarely induces mucous cell metaplasia in pathogen-free rats [20, 58] whereas mucous cell metaplasia/hyperplasia may be induced in non-pathogen-free rats. This difference may be best explained by priming of the middle ear mucosa by pathogens prior to ETO.

 This notion is further supported by the observation in cats that mucous cell metaplasia/hyperplasia with thick effusion in the middle ear cavity is readily induced by ETO alone (unpublished data at the Otopathology Lab, University of Minnesota). The reason for this appears to be that the middle ear of cats is frequently colonized with bacteria from the upper respiratory tract due to their relatively short Eustachian tube. It has been shown, on one hand, that pneumococcus-induced middle ear infection in rats results in higher goblet cell numbers compared with *H. influenzae*-induced middle ear infection.

 On the other hand, multiple challenges with pneumococcus in the middle ear induced more goblet cells than a single challenge in the middle ear of pneumococcus [20], suggesting that repeated middle ear pneumococcal infections or a long-term exposure to pneumococcal cell envelope are important determinants for the development of mucous cell metaplasia/hyperplasia in OM. In these experiments, it has been noted that bacterial remnants may remain in the middle ear cavity despite the absence of viable bacteria, similar to the human setting where bacterial cell envelope components were present in the middle ear cavity although bacterial cultures were sterile [59, 60].

## 5. Triggers of Mucous Cell Metaplasia/Hyperplasia

 It is well accepted that middle ear infection triggers mucous cell metaplasia. Experimentally, we and others have shown that bacterial infection or cytokine challenge of middle ear mucosa results in mucous cell metaplasia/hyperplasia [20, 24, 37, 58, 61–63]. Clinically, chronic OM with mucoid effusion is frequently preceded by upper respiratory tract infection [19]. An inflammatory cascade of events typically activates inflammatory cells, including but not limited to eosinophils, lymphocytes, and mast cells. It causes an immune response.

 This inflammatory reaction not only mediates expression of the mucin genes such as MUC5B but also mucous cell metaplasia/hyperplasia via an inflammatory mediator-dependent mechanism [5]. The middle ear epithelial mucosa of rodents usually contains few mucous cells as judged by histology or whole mount middle ear mucosa. Many inflammatory mediators that cause mucous cell metaplasia/hyperplasia in OM were originally defined in rodents [20, 58] and subsequently verified in humans [64].

**Figure 7:**
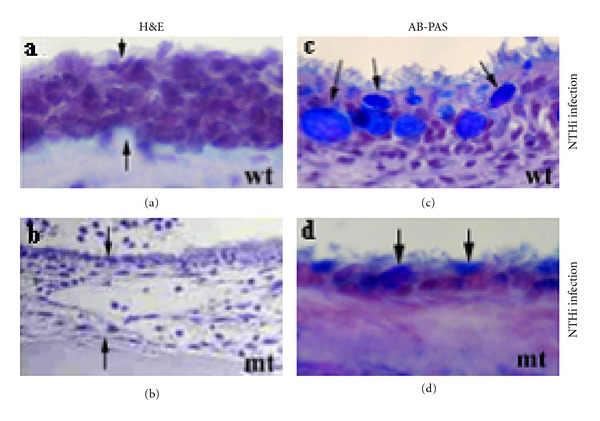
The middle ear mucosa in the wildtype (wt) mice responds to the challenge of nontypable *H. influenzae* (NTHi) and increases its mucous cell number (c). The middle ear mucosa in the interleukin 10 (IL-10) knockout mutant (mt) mice exhibits fewer mucous cells in response to NTHi infection (d). Arrows pointing to mucous cells.

 Cytokines are well known to be involved in mucous cell metaplasia/hyperplasia, especially proinflammatory cytokines and T-helper 2 (Th_2_) subset-derived cytokines. These cytokines include TNF*α* [37, 58, 65, 66], IL-13 [67–69], IL-10 [70], IL-8 [71], IL-9 [72], IL-4 [73], and so forth. Loss of cytokines in mice such as IL-10 results in reduced mucous cell metaplasia/hyperplasia in the middle ear of mice although it does not completely deplete the mucous cell population (Figure 7). It is noted that IL-9, IL-8, and IL-4 overexpression in the trachea increases mucous cell metaplasia/hyperplasia, but knockout of IL-9 did not affect the development of allergen-induced mucous cell metaplasia/hyperplasia [74]. The role of Th_2_ lymphocytes in mucous cell metaplasia/hyperplasia was determined by animal models in which marked mucous cell metaplasia/hyperplasia occurs in mice that received Th_2_ cells but not in mice that received Th_1_ cells [75].

 In T-, B-, and mast cell-deficient mice, reconstitution of CD4^+^ cells restores allergen-induced airway hypersensitivity, allergic inflammation, and mucous cell metaplasia/hyperplasia [76]. It suggests that mucous cell metaplasia/hyperplasia is dependent upon a subset of CD4^+^ T cells (regulatory, natural killer, or cytotoxic T cells). Possibly, it is a subpopulation of Th_2_ cells and their cytokines that mediate allergen-induced mucous cell metaplasia/hyperplasia. Theoretically, inflammatory mediators that strengthen Th_2_ cell functions and increase expression of Th_2_ cytokines are involved in mucous cell metaplasia/hyperplasia in the clinical setting of allergy. However, this does not necessarily mean that Th_2_ cytokines are indispensable in the inflammatory setting.

 Asthma is also known to trigger mucous cell metaplasia/hyperplasia in the respiratory tract. Typically, in asthma patients, mucous cell metaplasia/hyperplasia in peripheral airways is consistent pathologic characteristics of bronchial asthma [77]. It follows a similar paradigm as seen in bacterial infection; that is, systemic sensitization to allergens followed by repeated exposure to allergens causes an allergic inflammation. This reaction results in Th_2_ cytokine release and subsequent mucous cell metaplasia/hyperplasia in the respiratory tract mucosa [78, 79]. However, whether this is true in the middle ear epithelium under the condition of inflammation remains to be elucidated.

## 6. Signaling Pathways in Mucous Cell Metaplasia/Hyperplasia

It has been well established that bacterial signaling at mucosal surfaces processes that affect glycosylation in the literature [80, 81]. There may be more than one pathways involved in mucous cell metaplasia/hyperplasia of OM because of the importance of mucous cells in physiology and innate immune defense against infections. As described above, one well-recognized pathway is middle ear infection→inflammatory cell infiltration→cytokine production→mucous cell metaplasia/hyperplasia [5, 58]. Whether there is an inflammatory cell-independent pathway for mucous cell metaplasia in the middle ear was not known until our recent studies showed that PGPS incubation with mouse middle ear epithelial cells for 2 weeks *in vitro* led to the development of mucous-like cells in a cell culture system (referred to as Figure 9). This suggests that there are inflammatory cell-dependent and -independent mucous cell developmental pathways.

How mucous cells grow and differentiate in such a cell culture system is unclear. However, this provides an excellent model for the studies of mucous cell development. What would be the receptors and signal molecules that mediate the development of mucous cells? Firstly, TLRs are thought as the ones that trigger the signaling pathway because they specialize in recognition of bacterial pathogen-associated molecular pattern (PAMP). Indeed, TLR2 mediates Gram^+^ bacteria and their metabolites for activation of host cells [82]. TLR2 is expressed in mouse middle ear epithelial cells, and PGPS upregulates the expression of TLR2 [82].

Pneumococcus has been shown to bind to polymeric immunoglobulin A receptor (pIgAR) or platelet-activating factor receptor (PAFR) and enters nasopharyngeal epithelial cells through pIgAR [83] and lung epithelial cells through PAFR [84], but these receptors do not appear to activate host cells.

What would be the post-NF-*κ*B signal transduction cascade in terms of mucous cell development? It is unknown at the moment. Recent study indicates that Id1 is upregulated by pneumococcus [24]. In transgenic mice, Id1 overexpression in mice resulted in adenoma (mucous cell dysplasia) in the intestinal mucosa [85]. Transfection of mouse middle ear epithelium *in vivo* with Id1 for a week causes the proliferation of middle ear epithelial cells including mucous cells [86]. Transfection of middle ear epithelial cells with Id1 for two weeks followed by ETO for four weeks (a two-step method for induction of middle ear effusion) causes mucoid effusion in the middle ear cavity of rats [86].

**Figure 8:**
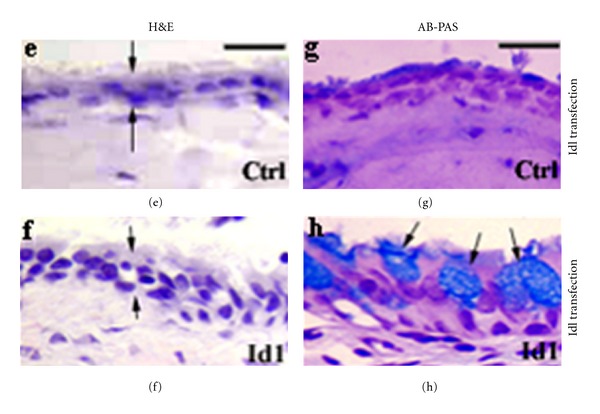
Id1 induces the proliferation of middle ear epithelial cells ((f), H&E stain) and mucous cells (arrows, (h), AB-PAS stain) compared with empty vector controls ((e), H&E, (g), AB-PAS). Note that the thickening of the middle ear epithelium is increased in Id1-transfected animals ((f), between arrows) compared with empty vector-transfected controls (e).

It is generally accepted that withdrawal from cell cycle is a prerequisite for cellular differentiation. It has been observed that Id1 has a dual effect on middle ear epithelial cell proliferation. Within the first 24 h of transfection with Id1, rats had increased DNA synthesis, cell cycle progression, and cell counts. After 24 h, however, these cells had decreased DNA synthesis, cell cycle progression, and cell counts and downregulated the mucin promoter activities. Correspondingly, transfection of the middle ear mucosa with Id1 increased the epithelial cell number and mucous cell counts (Figure 8).

**Figure 9:**
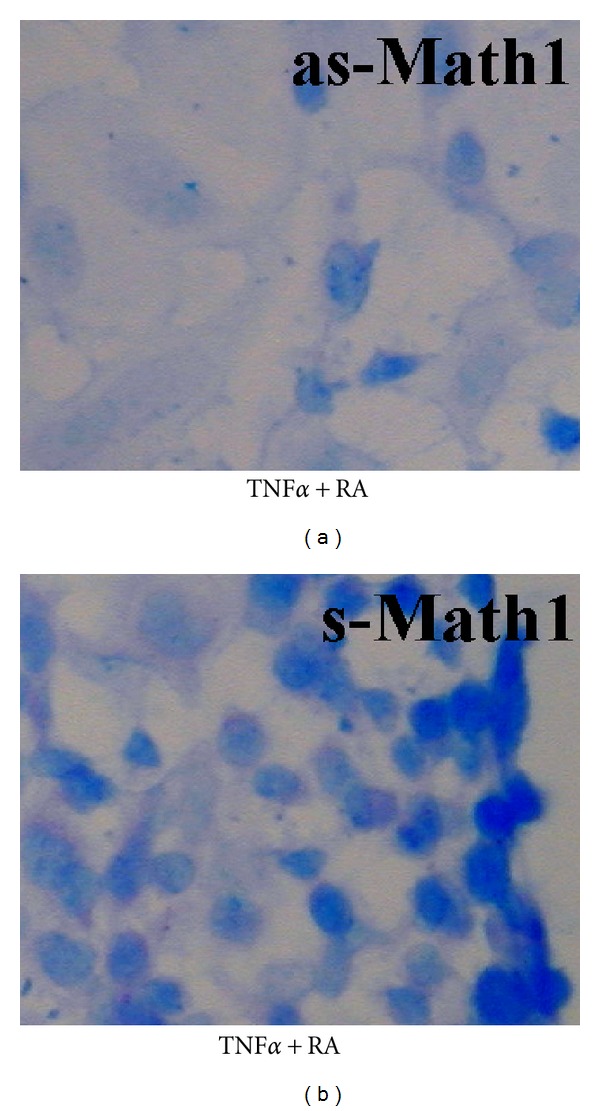
Math1 plays an important role in the differentiation of mucous-like cells. (a) With Math1 being knocked down by antisense-Math1 (as-Math1), AB-PAS-positive cells are obviously reduced in cultured mouse middle ear epithelial cells. (b) With Math1 being upregulated by sense-Math1 (s-Math1), AB-PAS-positive cells are remarkably increased. RA, retinoid acid; TNF*α*, tumor necrotic factor alpha.

Recent studies demonstrated that this process was associated with an increased expression of the *Math1* gene [86], suggesting that *Math1* plays an important role in the differentiation of mucous cells. Indeed, Math1 potentiated the expression of mucins *in vitro* (Figure 9).

*Math1* belongs to one of the helix-loop-helix (HLH) families and can form dimers with Id proteins and antagonize the effects of Id proteins, thus resulting in cellular differentiation. In referenced studies, *Math1* is required for the differentiation of progenitor cells into goblet cells in the intestinal epithelium, and the loss of the *Math1* gene leads to the depletion of goblet cells in the gut without affecting enterocytes [87]. Thus, it is possible that the Id1 and *Math1*, respectively, regulate mucous cell metaplasia in OM; Id proteins trigger proliferation of progenitor mucous cells, and *Math1* is responsible for the subsequent mucous cell differentiation. This notion is supported by *in vivo* studies in which overexpression of Id1 in the middle ear of mice caused epithelial cell hyperplasia and mucous cell metaplasia [86] whereas overexpression of Math1 in the middle ear of mice increased the mucous cell population [86].

On the basis of *Math1*, there are other factors which are important in the differentiation of mucous cells. As discussed above, multiple signaling pathways are involved in the differentiation of mucous cells. These signaling pathways are linked to inflammatory cytokines and/or physiological factors. There are many cytokines/chemokines/growth factors, as mentioned above, especially those Th_2_ cytokines that are frequently linked to the differentiation of mucous cells. In general, factors that are essential for the differentiation of mucous cells include fundamental transcription factors such as *Math1*, proinflammatory mediator such as TNF*α*, and epithelial cell differentiation factors such as retinoid acid. Experimentally, the addition of these three factors to cultured middle ear epithelial cells induces the differentiation of mucous-like cells *in vitro* (referred to as Figure 9).

In addition to *Math1,* other genes such as RELM-beta/FIZZ2 and gob5 are also relevant [88, 89].

Mucous cell metaplasia remains to be a great challenge for clinicians and researchers. There are no effective means of cure and prevention due to the lack of knowledge about the pathogenic mechanism of mucous cell metaplasia/hyperplasia in OM. Identification of the molecular pathways that mediate mucous cell metaplasia will not only facilitate the understanding of OM pathobiology but will also help develop effective means of prevention and treatment of chronic OM.

## 7. Future Directions

1. Origin of mucous cells in the middle ear mucosa: it is generally believed that all the epithelial cell types, goblet cells, and ciliated cells, in the middle ear mucosa, originate from middle ear epithelial stem cells. Unfortunately, nothing is known about the origin of epithelial stem cells in the middle ear mucosa. Many efforts and endeavors need to be placed in this new area in order to understand how mucous cells grow, proliferate, and differentiate into the terminal mucous cells in the middle ear setting.
2. Initial triggers of mucous cell differentiation: mutant mice provide a chance to identify the genes that are involved in the initial differentiation of mucous cells. As mentioned above, Math1 may serve as a trigger for initial differentiation of mucous cells. To this end, Math1 conditional knockout mutant mice are needed to prove whether Math1 is essential for mucous cell metaplasia/hyperplasia in the middle ear mucosa. If so, what is the next gene that pushes the initial differentiation of mucous cells further toward the terminal differentiation of mucous cells. In the intestine, GFI1 and SPDEF are needed [90]. It is unknown whether this is true to the middle ear.
3. Therapeutic agents for blockage of mucous cell metaplasia/hyperplasia: after identification of the mucous cell differentiation genes, otological scientists should be able to study and identify agents that can block mucous cell metaplasia/hyperplasia based upon the understanding of the molecular mechanism of mucous cell metaplasia/hyperplasia. Much work needs to be done in this particular area for identification of effective agents to stop mucous cell metaplasia/hyperplasia in OM.
